# Sagittal suture morphological variation in human archaeological populations

**DOI:** 10.1002/ar.24627

**Published:** 2021-04-05

**Authors:** Olivia Cheronet, Abigail Ash, Alexandra Anders, János Dani, László Domboróczki, Eva Drozdova, Michael Francken, Marija Jovanovic, Lidija Milasinovic, Ildiko Pap, Pál Raczky, Maria Teschler‐Nicola, Zdeněk Tvrdý, Joachim Wahl, Gunita Zariņa, Ron Pinhasi

**Affiliations:** ^1^ Department of Evolutionary Anthropology University of Vienna Vienna Austria; ^2^ Department of Archaeology University of York York UK; ^3^ Institute of Archeological Sciences, Eötvös Loránd University Budapest Hungary; ^4^ Déri Museum Debrecen Hungary; ^5^ István Dobó Castle Museum Eger Hungary; ^6^ Department of Experimental Biology, Section of Genetics and Molecular Biology, Laboratory of Biological and Molecular Anthropology Faculty of Science, Masaryk Univerzity Brno Czech Republic; ^7^ Osteology, State Office for Cultural Heritage Baden‐Wuerttemberg Constance Germany; ^8^ Museum of Vojvodina Novi Sad Vojvodina Republic of Serbia; ^9^ National Museum of Kikinda Kikinda Republic of Serbia; ^10^ Department of Anthropology Hungarian Natural History Museum Budapest Hungary; ^11^ Department of Anthropology Natural History Museum Vienna Vienna Austria; ^12^ Anthropos Institute, Moravian Museum Brno Czech Republic; ^13^ Institut für Naturwissenschaftliche Archäologie Abteilung Paläoanthropologie, University of Tübingen Tübingen Germany; ^14^ University of Latvia, Institute of Latvian History Riga Latvia

**Keywords:** archaeology, morphology, quantification, sagittal suture

## Abstract

Cranial sutures join the many bones of the skull. They are therefore points of weakness and consequently subjected to the many mechanical stresses affecting the cranium. However, the way in which this impacts their morphological complexity remains unclear. We examine the intrinsic and extrinsic mechanisms of human sagittal sutures by quantifying the morphology from 107 individuals from archaeological populations spanning the Mesolithic to Middle ages, using standardized two‐dimensional photographs. Results show that the most important factor determining sutural complexity appears to be the position along the cranial vault from the junction with the coronal suture at its anterior‐most point to the junction with the lambdoid suture at its posterior‐most point. Conversely, factors such as age and lifeways show few trends in complexity, the most significant of which is a lower complexity in the sutures of Mesolithic individuals who consumed a tougher diet. The simple technique used in this study therefore allowed us to identify that, taken together, structural aspects play a more important role in defining the complexity of the human sagittal suture than extrinsic factors such as the mechanical forces imposed on the cranium by individuals' diet.

## 1 INTRODUCTION

The human cranium is composed of 21 bones that are all tightly interlinked from a young age. The junctions between these bones (known as sutures) are composed of fibrous connective tissues, which, as a consequence of their greater elasticity compared to bone, enable a certain amount of movement between the individual units and therefore a level of flexibility in global cranial shape. From the initial stages of cranial development through to old age, sutures across the cranium achieve variable degrees of closure and do so at various stages of development, at times sealing completely leading to sutural obliteration. The sequence in which the various cranial sutures close is a well‐documented developmental process which can be used to estimate the biological age of individuals based on their bony remains (a number of these methods have been evaluated by Meindl & Lovejoy, 1985, and Key, Aiello, & Molleson, 1994).

From a macroscopic visual observation of the skull, sutures appear as irregular lines marking the extent of individual bones. These junctions vary in appearance from linear partitions to complex interlinked protrusions (referred to as sinuosity). The morphology of this latter pattern is often described in the same terms used to describe wave functions (namely magnitude and frequency). Structurally, there are a number of ways in which the intricate morphology of sutures allows for several functions to be performed efficiently. Firstly, sinuosity will lock bones on either side into place, thereby counteracting potential shearing between them (Herring & Mucci, 1991; Jaslow, 1990). Additionally, it appears that although compressive forces are best withstood by high levels of sinuosity, tensile stresses can be accommodated by various types of sinuosity (Rafferty & Herring, 1999). Altogether, this reflects some of the variety of strain‐related influences affecting similar sutural properties (complexity).

While some correlation between age and suture closure patterns have been observed (Key et al., 1994; Meindl & Lovejoy, 1985), the causes of variability in sutural morphological complexity are less well understood. It is clear that in early cranial development, the trend is toward an increase in suture complexity as the many cranial bones fuse (Di Ieva et al., 2013). However, once this process is completed in early adulthood, variation in the sutures' morphology remains poorly characterized. In addition, as points of discontinuity in surface structure, sutures represent areas of flexibility between rigid and independent cranial bones and are therefore an important element in the distribution of mechanical stresses throughout the cranium (Curtis, Jones, Evans, O'Higgins, & Fagan, 2013; Herring & Teng, 2000). These same forces may also be fundamental in determining suture morphology (Byron et al., 2004; Byron, Segreti, Hawkinson, Herman, & Patel, 2018), although direct causality is difficult to establish.

As one of the major mechanical strains on the skull, mastication force (strongly influenced by the nature of foods being consumed) is implicated in the structuring of cranial sutures. These strains vary as a direct consequence of changes in the masticatory regimes of mammals (Burn et al., 2010; Byron, 2009; Katsaros, Kiliaridis, & Berg, 1994). However, whether increases in masticatory strains leads to increases in suture complexity (as measured, for example, by sinuosity) remains uncertain, with conflicting evidence available. Katsaros et al. (1994) and Byron (2009) found support for this in rats and Capuchin monkeys, respectively, whereas, in contrast, Burn et al. (2010) argue for a simplification of sutures with harder diets in pigs. Additionally, suture complexity has been found to increase with age (Wu, Liu, Zhang, Zhu, & Norton, 2007), potentially suggesting a cumulative effect of these mechanical strains. Furthermore, as each specific suture joins a unique pair of bones, with unique sets of functions, each suture will experience specific mechanical stress conditions and respond accordingly with a unique morphology (as quantified in the porcine skull by Rafferty & Herring, 1999). All these factors make cranial sutures an interesting, but complex, record of events during an individual's life.

Studies of the determinants of sutural morphology have predominantly been performed in animal models, with only a few in humans (Kanisius & Luke, 1994; Miura et al., 2009; Sholts & Wärmländer, 2012; Wu et al., 2007; Czarnetzki, 2015), as obvious ethical constraints make it particularly difficult to control for extrinsic factors in humans. Nevertheless, throughout (pre)history, the lifeways of human beings have been subject to systematic changes, including the introduction of new dietary habits and methods of food preparation, providing opportunities to hypothesize upon associated alterations in cranial morphology. Changing dietary habits may have led to the modification of cranial stresses (Paschetta, de Azevedo, Castillo, & Martınez‐Abadıas, 2010; Rando, Hillson, & Antoine, 2014), with some populations going as far as artificially modifying cranial shape (Tubbs, Salter, & Oakes, 2006). This has been observed to cause a sutural response (Gottlieb, 1978; Sanchez‐Lara, Graham, Hing, Lee, & Cunningham, 2007; Teschler‐Nicola & Mitteroecker, 2007). It is consequently of great interest to apply sutural research to a variety of archaeological populations with differing cultural practices.

In Europe, the lifeways of local human populations have undergone several major transitions, perhaps the most dramatic of which was the shift away from a nomadic hunter‐gatherer to a more sedentary agricultural subsistence around 8,000 BP (Bellwood, 2005). Along with a change in the animal and plant species consumed, this time period also saw the adoption of novel Neolithic technologies by European populations. In particular, the manufacture of pottery enabled the application of more advanced food preparation techniques and the broadening of dietary spectra to include substantial amounts of cooked, processed, and baked foodstuffs. Further technological advances in subsequent millennia led to the increasingly sophisticated use of metal ores to aid in the refinement of food preparation within the eponymous archaeological periods: the Copper Age, the Bronze Age, and the Iron Age. From the introduction of pottery to the development of metal tools, the trend toward increasing external processing of foodstuffs in European prehistory has precluded a reduction in the mechanical labor involved in mastication.

This study utilizes crania from a number of European pre‐industrial archaeological contexts, spanning the Mesolithic to Medieval period (9,300–1,250 BP), to determine the impact of intrinsic (positional variation within the suture and individual age at death) and extrinsic factors (cultural practices impacting thermal and non‐thermal food preparation methods and consequently masticatory requirements of the individuals) on the morphological complexity of the human sagittal suture.

## 2 MATERIALS AND METHODS

A sample of 107 skulls from central Europe and the Baltic region spanning the Mesolithic to the Middle Ages was used for this study (Table S1 includes a detailed list and Table 1 summary statistics of the individuals analyzed). Estimation of age at death and determination of biological sex for each skeleton was conducted using standard osteological methods and from observation of both the crania and the *ossa coxae*, where possible (Phenice, 1969; Ferembach et al., 1980; Brooks & Suchey, 1990; Buikstra & Uberlaker, 1994; Buckberry & Chamberlain, 2002).

**TABLE 1:** Summary statistics of the individuals included in this study. “*n*” indicates the sample size for each category. For each of the three indicators of suture morphology, the mean is given followed by the standard deviation in brackets

	Complexity index	Maximum extent	Crossings
*Age*			
Adolescent (*n* = 6)	3.376(±0.992)	0.271(±0.047)	44.167(±14.796)
Young adult (*n* = 21)	3.369(±0.741)	0.279(±0.073)	46.952(±9.255)
Young middle adult (*n* = 24)	3.822(±1.022)	0.362(±0.221)	47.458(±11.248)
Old middle adult (*n* = 21)	3.628(±1.237)	0.298(±0.158)	45.762(±9.879)
Old adult (*n* = 7)	3.222(±0.541)	0.256(±0.059)	54.286(±5.282)
Mature adult (*n* = 1)	3.134(±—)	0.189(±—)	46(±—)
*Sex*			
Male (*n* = 51)	3.348(±0.946)	0.32(±0.101)	47.059(±10.418)
Female (*n* = 51)	3.514(±1.019)	0.28(±0.172)	47.333(±9.779)
*Period*			
Mesolithic (*n* = 11)	2.685(±0.721)	0.263(±0.038)	47.091(±10.653)
Neolithic (*n* = 33)	3.547(±1.138)	0.296(±0.118)	45.364(±10.744)
Eneolithic (*n* = 7)	3.003(±0.681)	0.283(±0.053)	49.571(±5.255)
Bronze age (*n* = 21)	3.509(±0.927)	0.314(±0.123)	46.571(±9.271)
Iron age (*n* = 16)	3.351(±0.825)	0.267(±0.052)	48.063(±9.132)
Medieval (*n* = 19)	3.679(±0.924)	0.335(±0.253)	47.737(±11.883)
*Suture section*			
First quarter (*n* = 107)	3.009(±1.011)	0.171(±0.051)	13.972(±5.233)
Second quarter (*n* = 107)	3.987(±1.26)	0.236(±0.053)	11.561(±3.112)
Third quarter (*n* = 107)	3.514(±1.189)	0.217(±0.051)	10.689(±3.495)
Fourth quarter (*n* = 107)	3.825(±1.968)	0.249(±0.155)	10.962(±4.249)

Studying the sagittal suture was motivated by its position which enabled a simple and reproducible digitization method, and its long length which enabled it to be broken down in sections to investigate variation within the suture.

**FIGURE 1:**
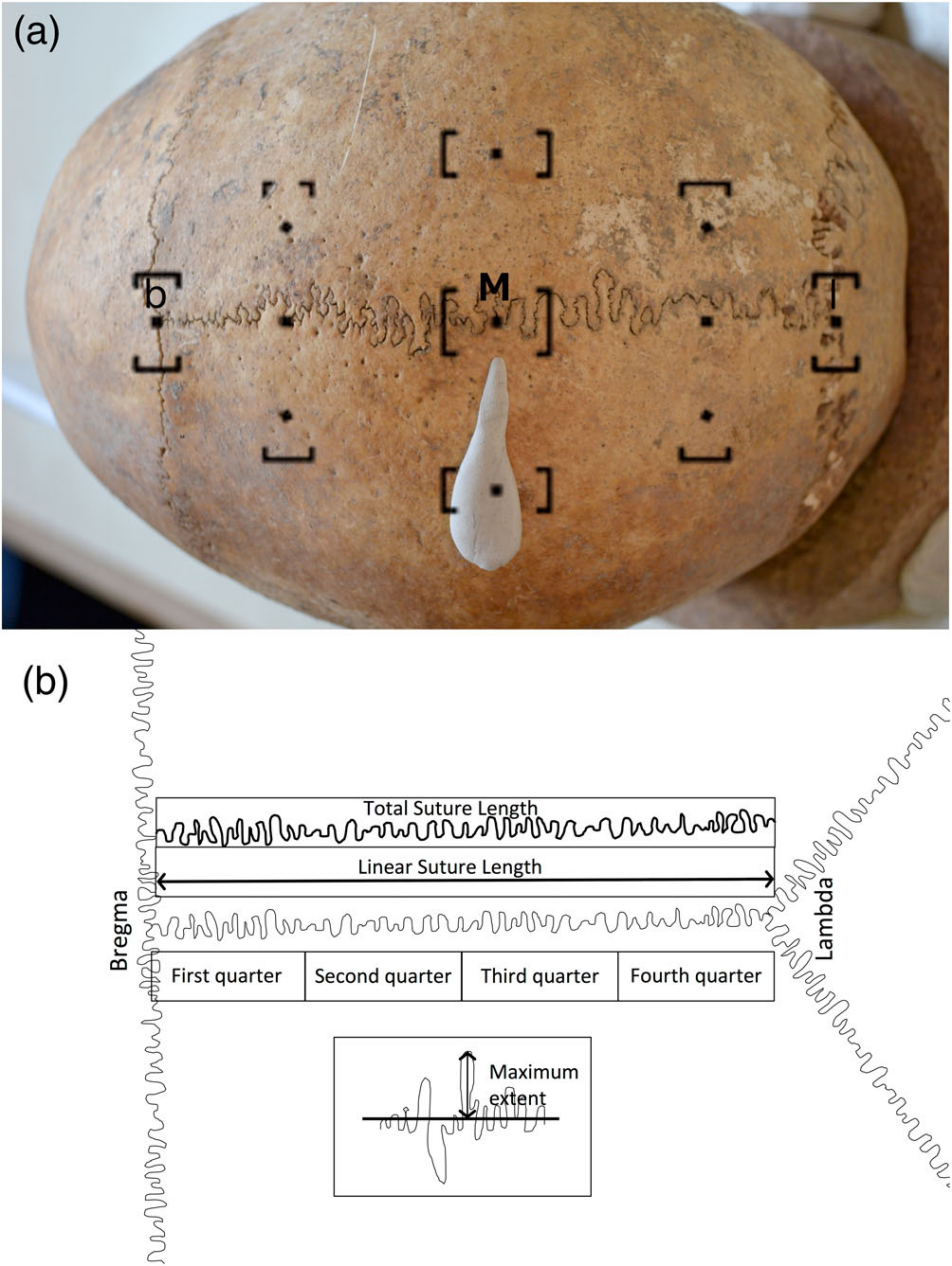
Quantification of the sagittal suture. (a) Standardized orientation for sagittal suture photography, as viewed through the camera. Point M, lambda (l) and bregma (b) are indicated. (b) Suture measurements and complexity indices calculated

All data were collected from standardized photographs of skulls taken at the site of collection storage. To avoid parallax errors as much as possible, the midpoint (calculated as half the suture's length as a chord on the surface of the skull) of the suture was identified (point M) and marked with a pointed piece of modeling clay. Following this, the viewfinder of a Nikon D3100 camera was used to obtain a standardized orientation. The middle focus point was aligned with point M, and the extreme left and right focus points with the start and end points of the sutures (bregma and lambda, respectively). See Figure 1a. for an exact view of the photographic protocol.

From an initially larger portfolio including all complete cranial vaults from all populations studied, 107 images (each depicting a unique cranium) were selected for inclusion within this study. Crania displaying substantial post mortem damage crossing the sagittal suture, as well as those with (near) complete obliteration of segments of the sagittal suture were excluded from the analysis. Furthermore, specimens with Wormian bones present at the junction of the sagittal and lambdoid sutures were only included when these bones took the form of small apical lambdoid ossicles, no larger than 2 cm^2^. All data relating to suture morphology were collected from this set of images.

Based on previous works, three indices were devised, thereby attempting to represent the sagittal suture morphology and complexity as thoroughly and simply as possible. The first was the “Complexity Index,” calculated by dividing the total length of the suture (obtained by tracing along the sinuous route of the suture) by the linear distance from bregma to lambda (henceforth linear suture length). This is a variation on the most frequently used measure of suture complexity (examples include Byron et al., 2004; Byron, 2009; Burn et al., 2010). The resulting index is independent of scale. This is also the case of the second measure: “Maximum Extent.” Here, the maximum lateral distance from the line joining bregma to lambda is divided by linear suture length. Finally, the only absolute value measured here is the number of times the suture crosses the linear suture length. These are all summarized in Figure 1b.

ImageJ (Abràmoff, Magalhães, & Ram, 2003) was used for the collection of measurements from images. Five sets of measurements were carried out for each individual. The first set for the entire suture, and the following four on each quarter of the suture, the subdivision of which was based on the linear suture length. As the curved nature of the cranial vault made it difficult to add a measurement scale that would apply to the entire length of the sagittal suture, all measurements were measured in terms of pixels on the suture photograph. For the skull as a whole, only the total suture length and the linear suture length were measured. The maximum distance from the midline and the number of crossings was inferred from the quarterly measurements. See Figure 1b for an illustration of suture measurements

All the statistical analyses were performed using the R (R Development Core Team, 2011) base package. As Shapiro Wilks tests suggested that the data obtained did not follow a normal distribution, the significance of the difference between all categories was assessed using Mann Whitney tests. The *p*‐values obtained for each of these are detailed in Table S2.

## 3 RESULTS

**FIGURE 2:**
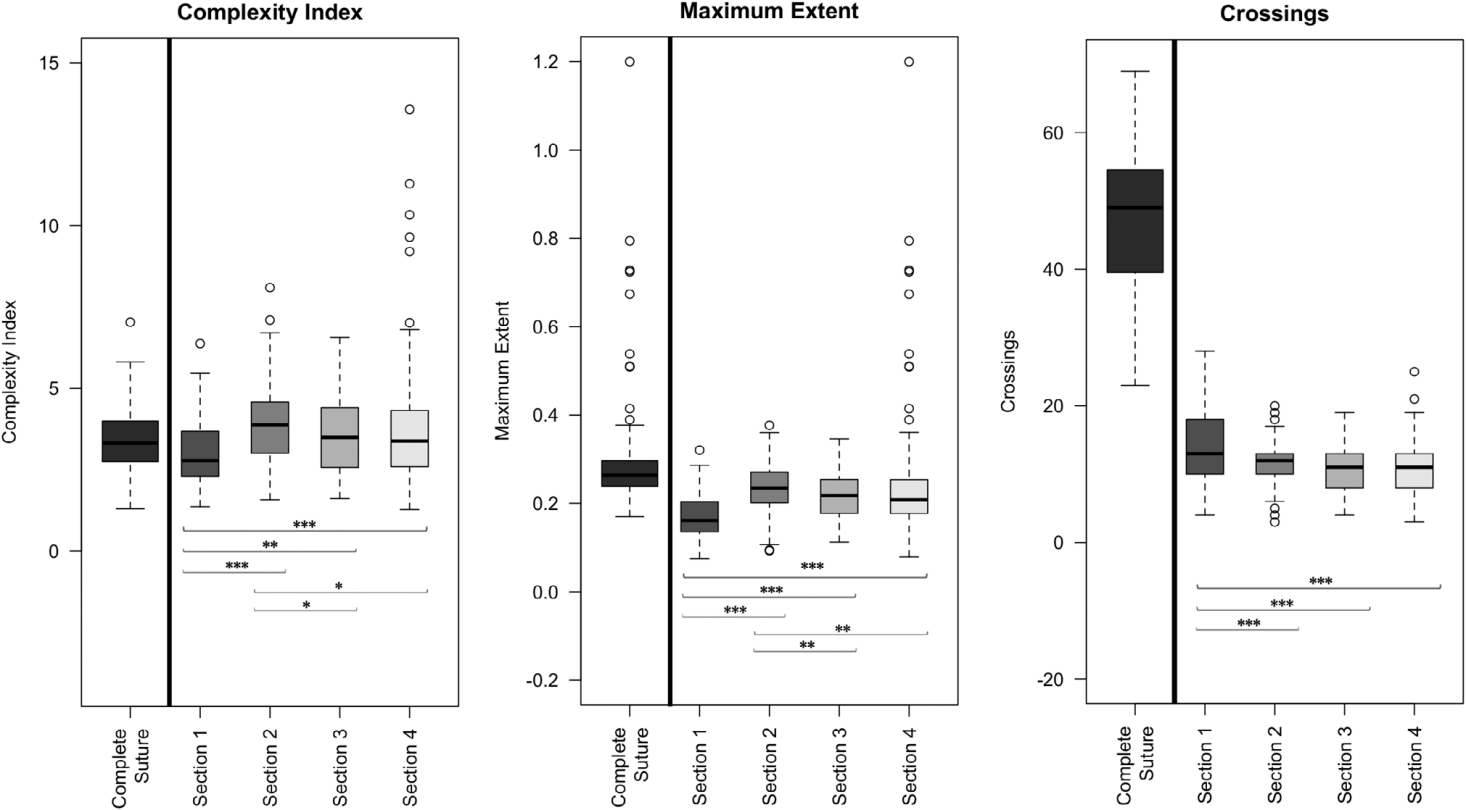
Variation of morphological complexity along the human sagittal suture. The significance of differences between sections was assessed by Mann Whitney test (*p value ≥ .05; **p value ≥ .01; ***p value ≥ .001)

Figure 2 illustrates the characterization of suture complexity along the whole of the sagittal suture. The high variability and large number of outliers in the complexity of the fourth (posterior‐most) section can probably be explained by the parallax error associated with its quantification through measurements on two‐dimensional images of this three‐dimensional structure. Keeping this in mind, the first section is that which differs the most from all other sections, in all three indices calculated, with a lower complexity, a narrower maximum extent and more crossings. Furthermore, although the number of crossings varies very little across the other three sections, section two has a higher complexity and extent than section three.

**FIGURE 3:**
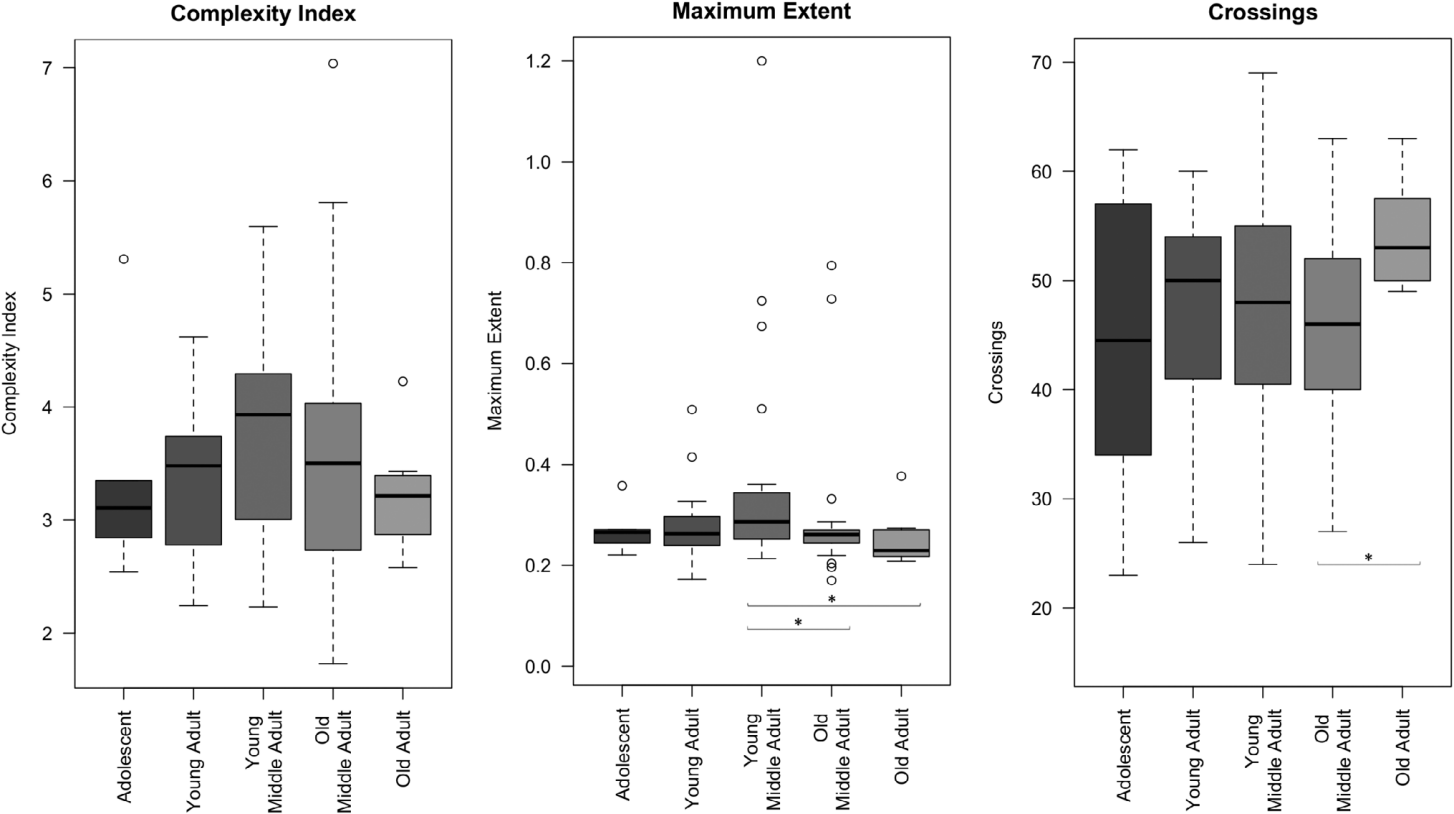
Variation of morphological complexity with individual age. The significance of differences between age groups was assessed by Mann Whitney test (*p value ≥ .05; **p value ≥ .01; ***p value ≥ .001)

**FIGURE 4:**
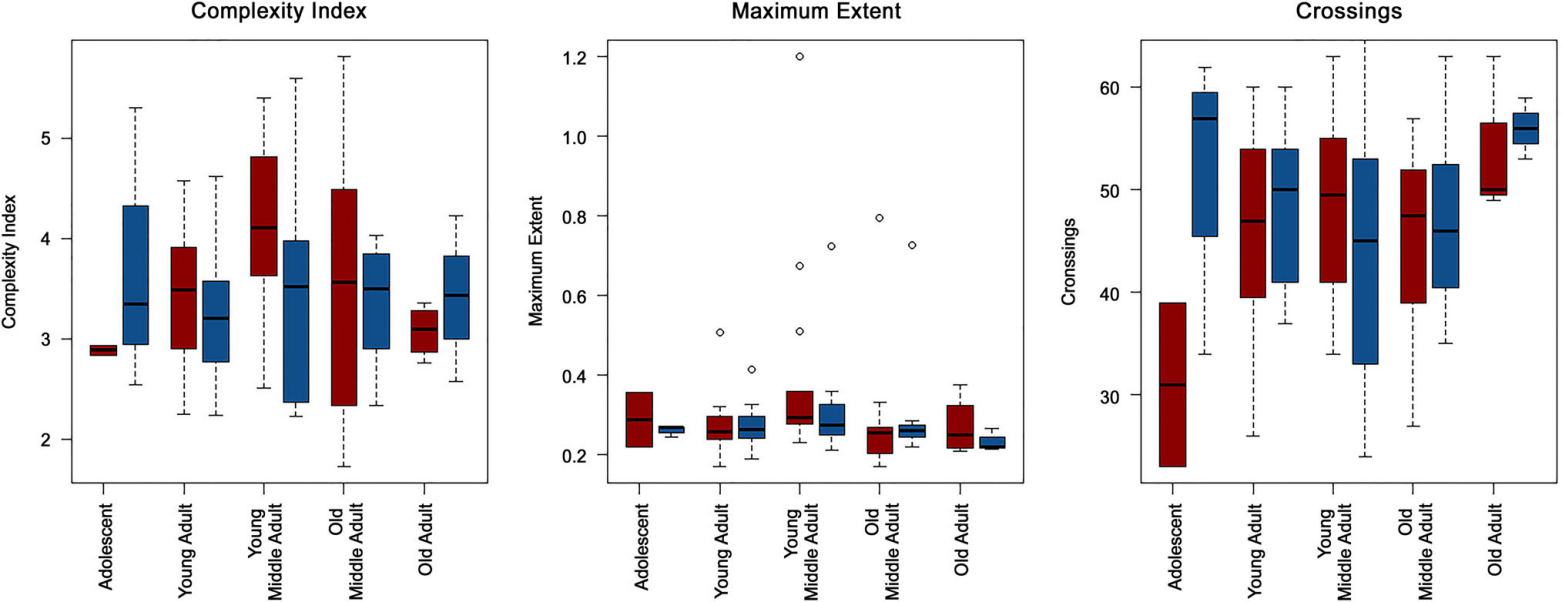
Variation of morphological complexity with individual age and separated by sex. The significance of differences between each category was assessed by Mann Whitney test (*p value ≥.05; **p value ≥ .01; ***p value ≥ .001). Blue bars represent males, and red bars represent females

In Figure 3, an analysis of complexity patterns across various age groups is depicted. Although statistical significance is weak, a peak in complexity index and maximum extent of sutures is observed in young middle adults (those individuals aged between 26 and 35 years at death). Conversely, the number of crossings appears to remain fairly constant throughout life with a significant increase in old adults (individuals aged 60 years and older at death). Furthermore, no sexual dimorphism is observed in these patterns (see Figure 4).

**FIGURE 5:**
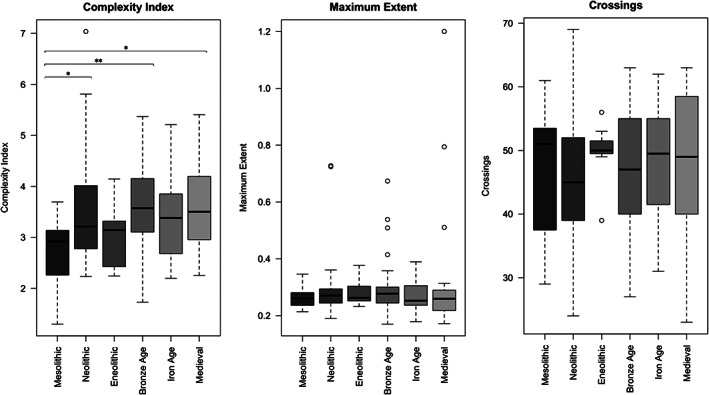
Variation of morphological complexity with archaeological periods. The significance of differences between periods was assessed by Mann Whitney test (*p value ≥ .05; **p value ≥ .01; ***p value ≥ .001)

Upon examination of sutural morphology by archaeological periods (Figure 5), the strongest pattern observed is one of reduced complexity in the Mesolithic group, which is statistically significant when compared to Neolithic, Bronze Age, and Medieval populations. In addition, one could argue for a very weak upward trend of the complexity index from the Mesolithic to the Medieval period. This however remains within the margins of error and may therefore be insignificant.

Taking the above‐discussed results all together, the main pattern emerging is one of complexity variation along the sagittal suture with a maximum complexity in section two. It is also interesting to note that an apparent peak in complexity is perhaps taking place in young middle adults.

## 4 DISCUSSION

The purpose of this study was to test the utility of sagittal suture morphological complexity as an indicator of changing masticatory stress regimes in human populations over 7,500 years of dietary and food preparation evolution, from the Mesolithic period to Medieval times. This was achieved through a quantitative characterization of morphological variation within the sagittal suture. Most studies of suture morphology assess sutures as single homogenous units. However, they can be long structures, as is the case of the human sagittal suture investigated here that regularly stretches over 15 cm. Consideration of separate sections within this suture clearly shows that sections within the sagittal suture respond to different influences and display variable morphological features over that 15 cm length.

In this work, observations of the morphology of the posterior‐most part are to be considered with caution as a result of parallax error. The curved nature of the human vault and the simple photographic set‐up used made it challenging to obtain two‐dimensional images with a clear focus throughout the full length of the sagittal suture. Nevertheless, when describing this structure in the same terms as those used for sinusoidal curves, the anterior‐most part of the sagittal suture tends to have a high frequency, but small amplitude. This was reversed for the second anterior‐most quarter of the suture. The third quarter had average values, and finally, similarly to the second quarter, the fourth and posterior‐most displayed a low frequency but high amplitude. This undoubtedly reflects variable constraints experienced along the suture.

Present studies of the factors impacting human sagittal suture morphology are scarce and rarely expressly seek out significant differences within the sagittal suture (Kanisius & Luke, 1994; Wu et al., 2007). There are a few more homologous studies in animals (Burn et al., 2010; Byron, 2009; Byron et al., 2004; Byron et al., 2018). A number of factors vary along this suture and may consequently explain this variation. Firstly, in its anterior part, the sagittal suture meets the coronal suture at a perpendicular angle. It may be theorized that this branching strongly changes the mechanical properties of this region. Sutures have long been assigned a strain distributing and dissipating function (Curtis et al., 2013; Jaslow & Biewener, 1995; Pritchard, Scott, & Girgis, 1956; Rafferty, Herring, & Marshall, 2003), though this opinion is not universal (Wang et al., 2012). The single sagittal suture branching laterally into two coronal sutures may permit a wider distribution of strain over the vault, allowing for a simpler morphology in the most anterior quarter. Furthermore, as the bulk of the mechanical strains in this region are tensile, resulting from the attachment of the masticatory muscles to the temporal bone (*temporalis*) and the nearby zygomatic arch (*masseter*), Rafferty and Herring (1999) suggests that a low sinuosity may be able to withstand such stresses efficiently. This is indeed also reflected in the work of Byron et al. (2018) who found no significant change in complexity with increasing dietary toughness on the anterior portion of murine sagittal sutures, but did find an increase in the posterior portion.

In the central part of the sagittal suture, the greater distance to the branching may result in it potentially having to withstand greater strains, possibly leading to the increase in complexity of the second quarter observed in the data. This then poses a new problem with the third quarter that decreases in sinuosity, which may however be related to the frequent presence of parietal foramina around this part of the suture. A wide magnitude in this area would cause an impossible overlap with the foramina, thereby constraining suture morphology regardless of mechanical strains. When the individuals with clear bilateral parietal foramina are isolated in this dataset, the lower complexity of the third quarter becomes more distinct, suggesting that foramina do impose some anatomical constraints. However, a small but apparent overall reduction in suture complexity in the third quarter may still be witnessed in crania without parietal foramina, indicating that this explanation is incomplete and that further research into this finding is needed.

Age‐related patterns of sagittal suture morphology were also observed. Although statistical signals are weak, young middle adults (corresponding to the ages of 26 to 35 years old at death) consistently achieve the highest levels of complexity (as measured by the complexity index and maximum extent), but older adults display the highest frequency of suture crossings (see Table S2). This could suggest that different factors may be at play in determining each of these morphological aspects. The relationship of suture morphology and age has been subject to some work. Wu et al. (2007) have suggested that suture complexity increases with age in humans, particularly until adolescence, and much more slowly thereafter. Conversely, by studying porcine sutures, Sun, Lee, and Herring (2004) found a decrease in sutural complexity with age associated with changing strain patterns. The data presented here suggests a slightly longer period of increase in complexity than Wu et al. (2007) followed by a regression to simpler sutures in later life. Maximum complexity appears to be reached in young middle adults, an age category still likely to represent the peak of muscular strength. Whether this difference is a consequence of the study by Wu et al. (2007) being based on modern, post‐industrial crania, whereas the here‐presented sample is based on pre‐industrial populations, and therefore shaped by masticatory functions performed on substantially different dietary items, is unclear.

In addition to the age of the individuals studied, the cultural context in which they lived also showed a significant relationship with complexity of the sagittal suture. Indeed, Mesolithic individuals display a generally lower suture complexity than those of other periods represented here (see Table S2). The transition from the Mesolithic to the Neolithic is one of the most important changes in human prehistory with a drastic change in lifeways from a hunting and gathering to a farming based subsistence. This is believed to be associated with a reduction in dietary toughness, which has been reflected in overall cranial morphology (Carlson & Van Gerven, 1977; González‐José et al., 2005; Paschetta et al., 2010; Pinhasi, Eshed, & Shaw, 2008). Aside from an overall cranial gracilization, there is also a notable brachycephalization of crania, whereby vaults became shorter and broader rather than elongate (Carlson & Van Gerven, 1977; Cheronet, Finarelli, & Pinhasi, 2016; Lalueza‐Fox, 1996).

Under the assumption of sutural complexity being dictated by the magnitudes of mechanical strains on the bone, the lower Mesolithic complexity observed here is surprising, as a decrease in dietary toughness would be expected to lead to a corresponding general decrease in sutural complexity of crania from the Neolithic and later periods (Byron, 2009; Katsaros et al., 1994; Wu et al., 2007), as well as more specifically in sinuosity (Byron et al., 2004; Kanisius & Luke, 1994). However, as the human cranium is a complex system, it is likely that the morphological changes toward a more gracile cranium associated with the transition to an agricultural life will also have played a significant role in responding to this modification in strain regime. Indeed, it can plausibly be hypothesized that a longer dolicocephalic skull, such as that of Mesolithic individuals, would be more efficient at distributing strains. Furthermore, within the sutures themselves, there are aspects other than complexity that may also change in response to changes in stress regimes. These include suture morphological irregularity (Liu et al., 2017) and beveling of sutures, as well as non‐sutural changes like a decrease in temporal area (Byron, Maness, Yu, & Hamrick, 2008) and a rise in the flexibility of the connective tissue within the suture (Byron et al., 2004). Indeed this soft tissue is fundamental in determining the ultimate morphology of the suture (Khonsari et al., 2013), but this cannot be assessed from archaeological contexts, where we rely solely on surviving hard tissue.

Although changes in dietary habits within later archaeological periods appear not to have played a significant part in explaining suture morphology, extending the study to later populations would be valuable. The fast and intense industrialization of the past 250 years has had a profound role in the modification of human lifeways, including diet (Cordain et al., 2005), and may therefore have had a stronger, and more visible, impact on suture morphology.

A number of studies have linked sutural complexity with dietary hardness in non‐human species (Burn et al., 2010; Byron, 2009; Byron et al., 2004; Byron et al., 2018; Kanisius & Luke, 1994; Katsaros et al., 1994; Kiliaridis, 1995; Rafferty & Herring, 1999; Sun et al., 2004). This has been supported experimentally, where the sagittal suture of animals fed on a tougher diet were observed to increase in sinuosity (Byron et al., 2018), widen (Katsaros et al., 1994) and thicken (Burn et al., 2010). However, neither of these latter two studies has reported a difference in sinuosity. A mechanically more demanding diet has been associated with higher levels of sinuosity in a robust species of Capuchin monkeys (Byron, 2009). One of the fundamental differences between these studies, however, is the scale: whereas the former two measure intra‐specific variation (and consequently phenotypic plasticity and bone remodeling), the latter takes an inter‐specific approach. Therefore, it could be hypothesized that observed differences in sinuosity between Capuchin species may be the result of phylogenetic distance. Conversely, plastic adjustments, although occasionally in the shape of increased sinuosity (as shown in Byron et al., 2018), can take the shape of more subtle changes including suture widening and thickening, an aspect not measured here, but of great interest for future work.

More sensitive morphology quantification methods such as surface scanning, and CT‐scanning can improve the performance of finer‐scaled studies. Additionally, the only suture investigated here is the sagittal suture, which runs along the superior edge of the cranial vault. However, mastication has been suggested to only exert small amounts of strain on the human vault (Behrents, Carlson, & Abdelnour, 1978; Hillam, 1996; Herring, Rafferty, Liu, & Marshall, 2001; Lieberman, 1996; Lieberman, Krovitz, Yates, Devlin, & St Claire, 2004; Toro‐Ibacache, Zapata Muñoz, & O'Higgins, 2016) in proportion to the rest of the skull, namely the bones of the face, including the maxilla, zygoma and nasal bones. It may therefore be interesting to perform a similar study on other sutures in areas undergoing stronger strains (e.g., the zygomaticotemporal suture), and consequently potentially exhibiting morphological changes of a higher magnitude.

In addition, although the human skull develops along a general mammalian plan, growth of the cranial vault follows a unique trajectory to accommodate an early, rapid expansion of the brain. The human brain is large proportionally to body size when compared with that of other mammals and much of its growth occurs within the first 3 years of life (Peterson, Warf, & Schiff, 2018). Consumption of milk and highly processed weaning foods for much of this growth period may limit masticatory strains in human infants at a time when sutural morphology is most plastic. In the model organisms used in the various studies mentioned here (Burn et al., 2010; Byron, 2009; Byron et al., 2004; Byron et al., 2018; Kanisius & Luke, 1994; Katsaros et al., 1994; Kiliaridis, 1995; Rafferty & Herring, 1999; Sun et al., 2004), cranial growth likely happened for a proportionally longer period, during which the effects of variable masticatory strains could be incorporated in the structure. Diet‐associated sutural response in humans may therefore be a lot more subtle than in other mammals.

Although this discussion of determinants of sagittal suture morphology has focused on various types of mechanical constraints, genetic factors should not be ruled out. Indeed, in forensic studies, the shape of the zygomaxillary suture has been utilized as an indicator of ancestry (Gill, 1995). Furthermore, many molecular pathways have a demonstrated involvement in the determination of sutural morphology (Miura et al., 2009; Morriss‐Kay & Wilkie, 2005; Rice, Kim, & Thesleff, 1997; Slater et al., 2008). Nevertheless, geometric morphometric studies have established that environmental factors play a very significant role in their determination (Sholts & Wärmländer, 2012). These genetic patterns are therefore unlikely to be of major importance in the here presented study.

## 5 CONCLUSION

In this study, we investigated the morphological complexity of human sagittal sutures in a number of archaeological populations. Their cultural heterogeneity allowed us to assess the potential effect of differing masticatory regimes, such as that associated with the Mesolithic‐Neolithic transition, on sutural complexity. The decrease in cranial strains associated with the consumption of the softer, more heavily processed Neolithic diet appears to have resulted in an increase in the complexity index of the sagittal suture, contrary to the suggestions of previous studies. Nevertheless, this effect is quite small in comparison to that of intrinsic structural constraints associated with the various anatomical features of the human skull. However, the focus here was on the sagittal suture only. The application of the here‐described suture‐quantification methods on other cranial sutures in differing anatomical contexts will undoubtedly help toward the further clarification of the impact of both intrinsic and extrinsic factors on sutural complexity.

## AUTHOR CONTRIBUTIONS

**Abigail Ash:** Data curation; formal analysis; investigation; writing‐original draft; writing‐review & editing. **Alexandra Anders:** Resources; writing‐review & editing. **János Dani:** Resources; writing‐review & editing. **László Domboróczki:** Resources; writing‐review & editing. **Eva Drozdova:** Resources; writing‐review & editing. **Michael Franken:** Resources; writing‐review & editing. **Marija Jovanovic:** Resources; writing‐review & editing. **Lidija Milasinovic:** Resources; writing‐review & editing. **Ildiko Pap:** Resources; writing‐review & editing. **Pál Raczky:** Resources; writing‐review & editing. **Maria Teschler‐Nicola:** Resources; writing‐review & editing. **Zdeněk Tvrdý:** Resources; writing‐review & editing. **Joachim Wahl:** Resources; writing‐review & editing. **Gunita Zariņa:** Resources; writing‐review & editing. **Ron Pinhasi:** Conceptualization; funding acquisition; project administration; supervision; writing‐original draft; writing‐review & editing.

## CONFLICT OF INTEREST

The authors declare no potential conflict of interest.

## Supporting information


**Table S1** Raw data used for this study.

**Table S2** Results of the Mann–Whitney tests performed.

## Data Availability

All raw measurements used for this study are provided as a supplementary table. Raw images are available from the corresponding author by request.

## References

[ar24627-bib-0001] Abràmoff, D. M. D. , Magalhães, P. J. , & Ram, S. J. (2003). Image processing with ImageJ. Biophotonics International, 11, 36–42.

[ar24627-bib-0002] Behrents, R. G. , Carlson, D. S. , & Abdelnour, T. (1978). In vivo analysis of bone strain about the sagittal suture in *Macaca mulatta* during masticatory movements. Journal of Dental Research, 57, 904–908. 10.1177/00220345780570091401 102671

[ar24627-bib-0003] Bellwood, P. S. (2005). First farmers: The origins of agricultural societies. Malden, MA: Blackwell Pub.

[ar24627-bib-0004] Brooks, S. , & Suchey, J. M. (1990). Skeletal age determination based on the os pubis: A comparison of the Acsádi‐Nemeskéri and Suchey‐Brooks methods. Human Evolution, 5, 227–238. 10.1007/BF02437238

[ar24627-bib-0005] Buckberry, J. L. , & Chamberlain, A. T. (2002). Age estimation from the auricular surface of the ilium: A revised method. American Journal of Physical Anthropology, 119, 231–239. 10.1002/ajpa.10130 12365035

[ar24627-bib-0006] Buikstra, J. E. , & Ubelaker, D. H. (1994). Standards for data collection from human skeletal remains. Fayetteville: Arkansas Archeological Survey.

[ar24627-bib-0007] Burn, A. , Herring, S. , Hubbard, R. , Zink, K. , Rafferty, K. , & Lieberman, D. (2010). Dietary consistency and the midline sutures in growing pigs: Dietary consistency and the midline sutures. Orthodontics & Craniofacial Research, 13, 106–113. 10.1111/j.1601-6343.2010.01483.x 20477970 PMC3384692

[ar24627-bib-0008] Byron, C. D. (2009). Cranial suture morphology and its relationship to diet in Cebus. Journal of Human Evolution, 57, 649–655. 10.1016/j.jhevol.2008.11.006 19833377

[ar24627-bib-0009] Byron, C. D. , Borke, J. , Yu, J. , Pashley, D. , Wingard, C. J. , & Hamrick, M. (2004). Effects of increased muscle mass on mouse sagittal suture morphology and mechanics. The Anatomical Record, 279A, 676–684. 10.1002/ar.a.20055 15224409

[ar24627-bib-0010] Byron, C. D. , Maness, H. , Yu, J. C. , & Hamrick, M. W. (2008). Enlargement of the temporalis muscle and alterations in the lateral cranial vault. Integrative and Comparative Biology, 48, 338–344. 10.1093/icb/icn020 21669796

[ar24627-bib-0011] Byron, C. , Segreti, M. , Hawkinson, K. , Herman, K. , & Patel, S. (2018). Dietary material properties shape cranial suture morphology in the mouse calvarium. Journal of Anatomy, 233, 807–813. 10.1111/joa.12888 30298923 PMC6231163

[ar24627-bib-0012] Carlson, D. S. , & Van Gerven, D. P. (1977). Masticatory function and post‐Pleistocene evolution in Nubia. American Journal of Physical Anthropology, 46, 495–506. 10.1002/ajpa.1330460316 871152

[ar24627-bib-0013] Cheronet, O. , Finarelli, J. A. , & Pinhasi, R. (2016). Morphological change in cranial shape following the transition to agriculture across western Eurasia. Scientific Reports, 6, 33316. 10.1038/srep33316 27622425 PMC5020731

[ar24627-bib-0014] Cordain, L. , Eaton, S. B. , Sebastian, A. , Mann, N. , Lindeberg, S. , Watkins, B. A. , … Brand‐Miller, J. (2005). Origins and evolution of the Western diet: Health implications for the 21st century. The American Journal of Clinical Nutrition, 81, 341–354. 10.1093/ajcn.81.2.341 15699220

[ar24627-bib-0015] Curtis, N. , Jones, M. E. H. , Evans, S. E. , O'Higgins, P. , & Fagan, M. J. (2013). Cranial sutures work collectively to distribute strain throughout the reptile skull. Journal of the Royal Society Interface, 10, 20130584. 10.1098/rsif.2013.0584 PMC3730698 23804444

[ar24627-bib-0060] Czarnetzki, A. , & Kory W . (2015). Endo‐ and Ectocranial Suture Closure in Relation to Modifying Factors. Lebenswelten von Kindern und Frauen in der Vormoderne, PAST Paläowissenschaftliche Studien 4, (123–133). Berlin: curach bhán publications.

[ar24627-bib-0016] Di Ieva, A. , Bruner, E. , Davidson, J. , Pisano, P. , Haider, T. , Stone, S. S. , … Grizzi, F. (2013). Cranial sutures: A multidisciplinary review. Child's Nervous System, 29, 893–905. 10.1007/s00381-013-2061-4 23471493

[ar24627-bib-0017] Ferembach, D. (1980). Recommendations for age and sex diagnoses of skeletons. Journal of Human Evolution, 9, 517–549.

[ar24627-bib-0018] Gill, G. W. (1995). Challenge on the frontier: Discerning American Indians from Whites osteologically. Journal of Forensic Sciences, 40, 15384J. 10.1520/JFS15384J 7595322

[ar24627-bib-0019] González‐José, R. , Ramírez‐Rozzi, F. , Sardi, M. , Martínez‐Abadías, N. , Hernández, M. , & Pucciarelli, H. M. (2005). Functional‐cranial approach to the influence of economic strategy on skull morphology. American Journal of Physical Anthropology, 128, 757–771. 10.1002/ajpa.20161 16028224

[ar24627-bib-0020] Gottlieb, K. (1978). Artificial cranial deformation and the increased complexity of the lambdoid suture. American Journal of Physical Anthropology, 48, 213–214. 10.1002/ajpa.1330480215 637122

[ar24627-bib-0022] Herring, S. W. , & Mucci, R. J. (1991). In vivo strain in cranial sutures: The zygomatic arch. Journal of Morphology, 207, 225–239. 10.1002/jmor.1052070302 1856873 PMC2814820

[ar24627-bib-0023] Herring, S. W. , Rafferty, K. L. , Liu, Z. J. , & Marshall, C. D. (2001). Jaw muscles and the skull in mammals: The biomechanics of mastication. Comparative Biochemistry and Physiology. Part A, Molecular & Integrative Physiology, 131, 207–219. 10.1016/s1095-6433(01)00472-x 11733178

[ar24627-bib-0024] Herring, S. W. , & Teng, S. (2000). Strain in the braincase and its sutures during function. American Journal of Physical Anthropology, 112, 575–593. 10.1002/1096-8644(200008)112:4<575::AID-AJPA10>3.0.CO;2-0 10918130 PMC2813197

[ar24627-bib-0025] Hillam, R. (1996). Response of bone to mechanical load and alterations in circulating hormones. Bristol: University of Bristol.

[ar24627-bib-0026] Jaslow, C. R. (1990). Mechanical properties of cranial sutures. Journal of Biomechanics, 23, 313–321. 2335529 10.1016/0021-9290(90)90059-c

[ar24627-bib-0027] Jaslow, C. R. , & Biewener, A. A. (1995). Strain patterns in the horncores, cranial bones and sutures of goats (*Capra hircus*) during impact loading. Journal of Zoology, 235, 193–210.

[ar24627-bib-0028] Kanisius, P. H. , & Luke, D. A. (1994). Is the complexity of the human sagittal suture related to the size of the temporal muscle? International Journal of Anthropology, 9, 265–272.

[ar24627-bib-0029] Katsaros, C. , Kiliaridis, S. , & Berg, R. (1994). Functional influence on sutural growth. A morphometric study in the anterior facial skeleton of the growing rat. European Journal of Orthodontics, 16, 353–360. 10.1093/ejo/16.5.353 7805808

[ar24627-bib-0031] Key, C. A. , Aiello, L. C. , & Molleson, T. (1994). Cranial suture closure and its implications for age estimation. International Journal of Osteoarchaeology, 4, 193–207. 10.1002/oa.1390040304

[ar24627-bib-0032] Khonsari, R. H. , Olivier, J. , Vigneaux, P. , Sanchez, S. , Tafforeau, P. , Ahlberg, P. E. , … Calvez, V. (2013). A mathematical model for mechanotransduction at the early steps of suture formation. Proceedings of the Royal Society B, 280, 20122670. 10.1098/rspb.2012.2670 23516237 PMC3619497

[ar24627-bib-0033] Kiliaridis, S. (1995). Masticatory muscle influence on craniofacial growth. Acta Odontologica Scandinavica, 53, 196–202. 10.3109/00016359509005972 7572097

[ar24627-bib-0034] Lalueza‐Fox, C. (1996). Physical anthropological aspects of the mesolithic neolithic transition in the Iberian Peninsula. Current Anthropology, 37, 689–695.

[ar24627-bib-0035] Lieberman, D. E. (1996). How and why humans grow thin skulls: Experimental evidence for systemic cortical Robusticity. American Journal of Physical Anthropology, 101, 217–236. 8893086 10.1002/(SICI)1096-8644(199610)101:2<217::AID-AJPA7>3.0.CO;2-Z

[ar24627-bib-0036] Lieberman, D. E. , Krovitz, G. E. , Yates, F. W. , Devlin, M. , & St Claire, M. (2004). Effects of food processing on masticatory strain and craniofacial growth in a retrognathic face. Journal of Human Evolution, 46, 655–677. 10.1016/j.jhevol.2004.03.005 15183669

[ar24627-bib-0037] Liu, L. , Jiang, Y. , Boyce, M. , Ortiz, C. , Baur, J. , Song, J. , & Li, Y. (2017). The effects of morphological irregularity on the mechanical behavior of interdigitated biological sutures under tension. Journal of Biomechanics, 58, 71–78. 10.1016/j.jbiomech.2017.04.017 28457605

[ar24627-bib-0038] Meindl, R. S. , & Lovejoy, C. O. (1985). Ectocranial suture closure: A revised method for the determination of skeletal age at death based on the lateral‐anterior sutures. American Journal of Physical Anthropology, 68, 57–66. 10.1002/ajpa.1330680106 4061602

[ar24627-bib-0039] Miura, T. , Perlyn, C. A. , Kinboshi, M. , Ogihara, N. , Kobayashi‐Miura, M. , Morriss‐Kay, G. M. , & Shiota, K. (2009). Mechanism of skull suture maintenance and interdigitation. Journal of Anatomy, 215, 642–655. 10.1111/j.1469-7580.2009.01148.x 19811566 PMC2796787

[ar24627-bib-0040] Morriss‐Kay, G. M. , & Wilkie, A. O. M. (2005). Growth of the normal skull vault and its alteration in craniosynostosis: Insights from human genetics and experimental studies. Journal of Anatomy, 207, 637–653. 10.1111/j.1469-7580.2005.00475.x 16313397 PMC1571561

[ar24627-bib-0041] Paschetta, C. , de Azevedo, S. , Castillo, L. , & Martınez‐Abadıas, N. (2010). The influence of masticatory loading on craniofacial morphology: A test case across technological transitions in the Ohio valley. American Journal of Physical Anthropology, 18, 297–314. 10.1002/ajpa.21151 19902454

[ar24627-bib-0042] Peterson, M. , Warf, B. C. , & Schiff, S. J. (2018). Normative human brain volume growth. Journal of Neurosurgery. Pediatrics, 21, 478–485. 10.3171/2017.10.PEDS17141 29498607 PMC6212293

[ar24627-bib-0043] Phenice, T. W. (1969). A newly developed visual method of sexing the os pubis. American Journal of Physical Anthropology, 30, 297–301. 10.1002/ajpa.1330300214 5772048

[ar24627-bib-0044] Pinhasi, R. , Eshed, V. , & Shaw, P. (2008). Evolutionary changes in the masticatory complex following the transition to farming in the southern Levant. American Journal of Physical Anthropology, 135, 136–148. 10.1002/ajpa.20715 18046779

[ar24627-bib-0045] Pritchard, J. J. , Scott, J. H. , & Girgis, F. G. (1956). The structure and development of cranial and facial sutures. Journal of Anatomy, 90, 73–86. 13295153 PMC1244823

[ar24627-bib-0046] R Development Core Team . (2011). R: A language and environment for statistical computing. Vienna: R Foundation for Statistical Computing.

[ar24627-bib-0047] Rafferty, K. L. , & Herring, S. W. (1999). Craniofacial sutures: Morphology, growth, and in vivo masticatory strains. Journal of Morphology, 242, 167–179. 10.1002/(SICI)1097-4687(199911)242:2<167::AID-JMOR8>3.0.CO;2-1 10521876 PMC2813870

[ar24627-bib-0048] Rafferty, K. L. , Herring, S. W. , & Marshall, C. D. (2003). Biomechanics of the rostrum and the role of facial sutures. Journal of Morphology, 257, 33–44. 10.1002/jmor.10104 12740894 PMC2819158

[ar24627-bib-0049] Rando, C. , Hillson, S. , & Antoine, D. (2014). Changes in mandibular dimensions during the mediaeval to post‐mediaeval transition in London: A possible response to decreased masticatory load. Archives of Oral Biology, 59, 73–81. 10.1016/j.archoralbio.2013.10.001 24169153

[ar24627-bib-0050] Rice, D. P. C. , Kim, H.‐J. , & Thesleff, I. (1997). Detection of gelatinase B expression reveals osteoclastic bone resorption as a feature of early calvarial bone development. Bone, 21, 479–486. 10.1016/S8756-3282(97)00182-8 9430236

[ar24627-bib-0051] Sanchez‐Lara, P. A. , Graham, J. M. , Hing, A. V. , Lee, J. , & Cunningham, M. (2007). The morphogenesis of wormian bones: A study of craniosynostosis and purposeful cranial deformation. American Journal of Medical Genetics. Part A, 143A, 3243–3251. 10.1002/ajmg.a.32073 18000970

[ar24627-bib-0052] Sholts, S. B. , & Wärmländer, S. K. T. S. (2012). Zygomaticomaxillary suture shape analyzed with digital morphometrics: Reassessing patterns of variation in American Indian and European populations. Forensic Science International, 217, 234.e1–234.e6. 10.1016/j.forsciint.2011.11.016 22154439

[ar24627-bib-0053] Slater, B. J. , Lenton, K. A. , Kwan, M. D. , Gupta, D. M. , Wan, D. C. , & Longaker, M. T. (2008). Cranial sutures: A brief review. Plastic and Reconstructive Surgery, 121, 170e–178e. 10.1097/01.prs.0000304441.99483.97 18349596

[ar24627-bib-0054] Sun, Z. , Lee, E. , & Herring, S. W. (2004). Cranial sutures and bones: Growth and fusion in relation to masticatory strain. The Anatomical Record. Part A, Discoveries in Molecular, Cellular, and Evolutionary Biology, 276, 150–161. 10.1002/ar.a.20002 14752854 PMC2813868

[ar24627-bib-0055] Teschler‐Nicola, M. , & Mitteroecker, P. (2007). Von künstlicher Kopfformung. In Historisches Museum der Pfalz Speyer (Hrsg.), Attila und die Hunnen (pp. 270–279). Stuttgart: Konrad Theiss Verlag.

[ar24627-bib-0056] Toro‐Ibacache, V. , Zapata Muñoz, V. , & O'Higgins, P. (2016). The relationship between skull morphology, masticatory muscle force and cranial skeletal deformation during biting. Annals of Anatomy, 203, 59–68. 10.1016/j.aanat.2015.03.002 25829126

[ar24627-bib-0057] Tubbs, R. S. , Salter, E. G. , & Oakes, W. J. (2006). Artificial deformation of the human skull: A review. Clinical Anatomy, 19, 372–377. 10.1002/ca.20177 16092127

[ar24627-bib-0058] Wang, Q. , Wood, S. A. , Grosse, I. R. , Ross, C. F. , Zapata, U. , Byron, C. D. , … Strait, D. S. (2012). The role of the sutures in biomechanical dynamic simulation of a macaque cranial finite element model: Implications for the evolution of craniofacial form. Anatomical Record (Hoboken), 295, 278–288. 10.1002/ar.21532 PMC3365596 22190334

[ar24627-bib-0059] Wu, X. , Liu, W. , Zhang, Q. , Zhu, H. , & Norton, C. J. (2007). Craniofacial morphological microevolution of Holocene populations in northern China. Chinese Science Bulletin, 52, 1661–1668. 10.1007/s11434-007-0227-8

