# Supplementary material for: Sagittal suture morphological variation in human archaeological populations
Source: Anat Rec (Hoboken). 2021 Apr 5;304(12):2811–22. doi: 10.1002/ar.24627 (PMC9291749; doi:10.1002/ar.24627)
Supplement: Supplementary file 2 — Table S2 Results of the Mann–Whitney tests performed. [file AR-304-2811-s002.docx]

|  |  |  |  |  |  |
| --- | --- | --- | --- | --- | --- |
| ***Age*** |  |  |  |  |  |
| *adolescent* | *young adult* | *young middle adult* | *old middle adult* | *old adult* | *mature adult* |
|  | 0.7547/0.9774/0.8152 | 0.3467/0.1914/0.6591 | 0.5124/0.5888/0.7704 | 0.8357/0.4452/0.2813 | --/--/-- |
| *young adult* | *young middle adult* | *old middle adult* | *old adult* | *mature adult* |  |
|  | 0.1313/0.1498/0.9455 | 0.6721/0.6721/0.6594 | 0.5329/0.2721/0.08845 | --/--/-- |  |
| *young middle adult* | *old middle adult* | *old adult* | *mature adult* |  |  |
|  | 0.3038/**0.03441**/0.6245 | 0.2342/**0.04277**/0.0977 | --/--/-- |  |  |
| *old middle adult* | *old adult* | *mature adult* |  |  |  |
|  | 0.4349/0.6781/**0.04084** | --/--/-- |  |  |  |
| *old adult* | *mature adult* |  |  |  |  |
|  | --/--/-- |  |  |  |  |
|  |  |  |  |  |  |
| ***Period*** |  |  |  |  |  |
| *Mesolithic* | *Neolithic* | *Eneolithic* | *Bronze Age* | *Iron Age* | *Medieval* |
|  | **0.02864**/0.4217/0.6254 | 0.4252/0.4252/0.8915 | **0.008327**/0.2381/1 | 0.08886/0.9035/0.8434 | **0.0124**/0.6719/1 |
| *Neolithic* | *Eneolithic* | *Bronze Age* | *Iron Age* | *Medieval* |  |
|  | 0.2926/0.9169/0.2848 | 0.5975/0.6987/0.6189 | 0.7758/0.5059/0.3645 | 0.4855/0.299/0.3418 |  |
| *Eneolithic* | *Bronze Age* | *Iron Age* | *Medieval* |  |  |
|  | 0.1553/0.7166/0.5398 | 0.3084/0.376/0.8669 | 0.08286/0.3946/0.8166 |  |  |
| *Bronze Age* | *Iron Age* | *Medieval* |  |  |  |
|  | 0.4573/0.2941/0.6785 | 0.7888/0.2154/0.5969 |  |  |  |
| *Iron Age* | *Medieval* |  |  |  |  |
|  | 0.2569/0.6121/0.8684 |  |  |  |  |
|  |  |  |  |  |  |
| ***Age and Sex*** |  |  |  |  |  |
| *male adolescent* | *female adolescent* |  |  |  |  |
|  | 0.8/1/0.4 |  |  |  |  |
| *male young adult* | *female young adult* |  |  |  |  |
|  | 0.6511/0.8078/0.5932 |  |  |  |  |
| *male young middle adult* | *female young middle adult* |  |  |  |  |
|  | 0.1083/0.3407/0.3955 |  |  |  |  |
| *male old middle adult* | *female old middle adult* |  |  |  |  |
|  | 0.9725/0.8094/0.7781 |  |  |  |  |
| *male old adult* | *female old adult* |  |  |  |  |
|  | 0.6286/0.6286/0.3725 |  |  |  |  |
| *male mature adult* | *female mature adult* |  |  |  |  |
|  | --/--/-- |  |  |  |  |
